# Increase of Compact Bone Thickness in Rat Tibia after Implanting MgO into the Bone Marrow Cavity

**DOI:** 10.3390/jfb5030158

**Published:** 2014-09-11

**Authors:** Håkan Nygren, Mobina Chaudhry, Stefan Gustafsson, Göran Kjeller, Per Malmberg, Kjell-Erik Johansson

**Affiliations:** 1 Department of Cell Biology and Medical Chemistry, Institute of Biomedicine, University of Gothenburg, P.O. Box 420, 405 30 Göteborg, Sweden; E-Mail: mobina.chaudhry@anatcell.gu.se; 2 Department of Applied Physics, Chalmers University of Technology, 412 96 Göteborg, Sweden; E-Mail: stefan.gustafsson@chalmers.se; 3 Department of Oral and Maxillofacial Surgery, Institute of Odontology, University of Gothenburg, P.O. Box 450, 405 30 Göteborg, Sweden; E-Mail: Goran.Kjeller@odontologi.gu.se; 4 National Center of Imaging Mass Spetrometry, Chalmers University of Technology, 412 96 Göteborg, Sweden; E-Mail: malmper@chalmers.se; 5 Elos Medtech, 540 16 Timmersdala, Sweden; E-Mail: kjell-erik.johansson@elosmedtech.com

**Keywords:** rat model, bone density, bone healing, bone remodeling, magnesium-oxide, ToF-SIMS, EDX.

## Abstract

The effect of implanting MgO paste into the bone marrow of rat tibia, was studied by light microscopy, time of flight-secondary ion mass spectrometry (ToF-SIMS), and environmental scanning electron microscopy (ESEM), and energy dispersive X-ray (EDX) analysis. After three weeks of implantation, the thickness of compact bone increased by 25% compared to sham-operated controls, while no effect was seen on the trabecular bone. In order to further elucidate the mechanism of the Mg-induced increase in bone mass, EDX and ToF-SIMS analysis of the bone samples was made at two weeks. At this time-point, no detectable difference in the thickness of the compact bone in Mg-treated and non-treated animals was observed. The Mg-content of the bone marrow and bone tissue of the Mg-exposed animals did not differ from that of sham-operated controls, implying that there are no traces of the implanted MgO when the mass of compact bone increases, between two and three weeks after surgery. The ratio of Mg/Ca content was higher in the bone of Mg-treated animals, indicating an altered structure of the bone mineral, which was confirmed by the ToF-SIMS analysis, showing increased levels of MgCO_3_, phosphate ions and CaF in the bone of MgO-exposed animals. Possible cellular activities behind the effect of MgO on compact bone thickness are discussed.

## 1. Introduction

Magnesium (Mg) ions are the second most abundant cellular cations and are known to participate in the regulation of cellular processes like cell cycle and cell differentiation [1], indicating a possible role in processes like bone healing and remodeling.

During the last decade, an increasing number of publications have appeared on the use of Mg as a resorbable biomaterial. One application is the use of Mg as a degradable stent which has been proven successful in animal experiments [2]. Another area of application is in orthopedics, where Mg has been demonstrated as a resorbable, biocompatible and bone promoting material [3]. Magnesium oxide has been shown to induce bone formation in experimental animal models and to stimulate bone formation *in vitro* [4,5,6].

Already, in 1924, Arthur A. Zierold published a study [7] “with the object to determine whether metal *per se*, when implanted in bone, exerts an influence other than that of any foreign body, and if so, whether there is a property common to many metals or varying with each individual metal”. The study included a large number of mature dogs, and the analysis of the outcome was made by X-ray and light microscopy.

The results of Zierolds’ study indicate that the healing of bone at metal implants resembles normal fracture healing of bone in that a callus of immature, woven bone is formed beneath the periosteal and endosteal layers, respectively. The amount of callus bone formed was found to vary with the individual implanted metals and was interpreted as a result of successful healing. The possibility of remodeling as a cause of variation in the amount of callus, was not considered at the time. Implanted Mg was shown to increase the amount of connective tissue (fibrosis) and callus-bone formed under the periosteal membrane. However, the lesion of implantation surgery did not heal, and new bone was not seen in contact with the implant, which was interpreted as retardation of bone healing. These findings were also later confirmed by McBride [8,9]. The information obtained from the studies by Zierold and McBride was used to develop a titanium device with a surface layer of magnesium (EP 1345637 B1, “Surface modification of implants for healing in bone and soft tissue”), The aim of the present study was to further elucidate resulting long-term effect of Mg on bone mass after implanting MgO into the bone marrow cavity.

## 2. Materials and Methods

The study was approved by the Ethical Review board at the University of Gothenburg.

### 2.1. Surgery

Pure MgO-powder—99.995 trace metal basis (Sigma-Aldrich^®^ Sweden AB, Kista, Sweden) was heat sterilized in closed glass containers in a pre-heated oven at 160 °C for 90 min and allowed to cool in the oven. The sealed containers were opened just a few minutes before use. All instruments were stored in 70% ethanol, using antiseptic surgery technique.

Male Sprague Dawley rats 200 g at arrival from Charles River, Holland were used throughout the study. The animals were anesthetized with Isofluran (Baxter Medical CO, Kista, Sweden). The calves were shaved and cleaned with iodine. An incision, approximately 2 cm in length, was made through the skin exposing the muscle layer. The anterior tibial muscle was retracted laterally in order to expose the tibial bone. A small hole, 1 mm in diameter, was drilled in the lateral face of the tibial bone, one third of a bone length inferior to the knee, using a low speed drill under extensive irrigation with sterile saline. Sterile MgO powder was mixed with sterile saline into a paste. The paste was packed into the drilled cavity in 7 rats. In 7 other rats, the drilled hole was left as sham control. The muscle was then used to cover the defect and, then, the skin was sutured with Suturamid 4-0 (Johnson and Johnson Intl; Brussels, Belgium). Pain relief, buprenorphine (Temgesic; Reckit and Coleman, Hull, UK; 0.05 mg/kg b.wt.) was given immediately after surgery subcutaneously. The animals showed normal behavior after surgery, and only one injection of Temgesic was administered.

The animals had access to food and water *ad libidum*. The rats were terminated at 2 or 3 weeks after the surgery through removal of the heart under general anesthesia. The respective tibial bone were dissected out and prepared for analysis.

### 2.2. Analysis

#### 2.2.1. Histology

The samples were fixed in 1% paraformaldehyde in phosphate-buffered saline (PBS) for 3 days. The bone samples were then decalcified for 2 weeks in 0.15 M ethylenediaminetetraacetic acid tetrasodium dehydrate (EDTA; Sigma-Aldrich^®^ Sweden AB) containing 0.5% paraformaldehyde. The medium was changed every third day. The samples were then left to Histolab Products (Göteborg, Sweden), for preparation by dehydration in graded series of ethanol, placed in xylene embedded in Histowax embedding medium at 60 °C. The samples were then cut and mounted on Superfrost Plus glass slides (Menzel-Gläser, Germany) and stained with hematoxylin and eosin and examined in a Zeiss Axioscope 2 microscope, equipped with an Axiocam ICc 1 digital camera (Zeiss, Jena, Germany).

#### 2.2.2. Environmental Scanning Electron Microscopy

The rat tibiae were immersed into absolute ethanol at −78 °C (dry ice). The bone tissue was substituted with alcohol for one week, warmed to room temperature, and cut with a diamond saw, using absolute ethanol as lubricating liquid, and rinsed carefully in absolute ethanol. The samples were left to dry at room temperature. An FEI Quanta 200 FEG ESEM operating at an accelerating voltage of 20 kV was used for imaging and chemical analysis. All images were acquired in the backscattered electron (BSE) imaging mode and at a pressure of 1 torr in the low vacuum region in order to avoid charging effects.

Energy dispersive X-ray (EDX) data was recorded using an Oxford EDX detector and spectra were evaluated with the INCA software (ETAS Group, Stuttgart, Germany).

ToF-SIMS analysis was performed with a TOF.SIMS 5 instrument (ION-TOF GmbH, Münster, Germany) using a Bi+ 3 cluster ion gun as the primary ion source was used and a 10 kV C_60_-source for sputtering. First, the whole bone region was sputter cleaned with C60^++^ (2.4 nA, 700 μm × 700 μm sputter area, Dose Density 3.06 × 10^13^) to remove surface contaminations. Then multiple (*n* = 5) regions at 105 μm × 105 μm from the edge of the bone marrow cavity into the cortical bone was analyzed with a pulsed primary ion beam (Bi+ 3, 0.31 pA at 25 keV, Dose density 1.12 × 10^11^) with a focus of approximately 2 μm and a mass resolution of *M*/Δ*M* = 5 × 10^3^ fwhm at m/z 500. All spectra were acquired and processed with the Surface Lab software (version 6.3, ION-TOF GmbH, Münster, Germany) and the ion intensities used for calculations were normalized to the total ion dose of each measurement. All spectra were calibrated internally to signals of [C]^+^, [CH]^+^, [CH_2_]^+^, [CH_3_]^+^ and [CaOH]^+^.

## 3. Results

**Figure 1:**
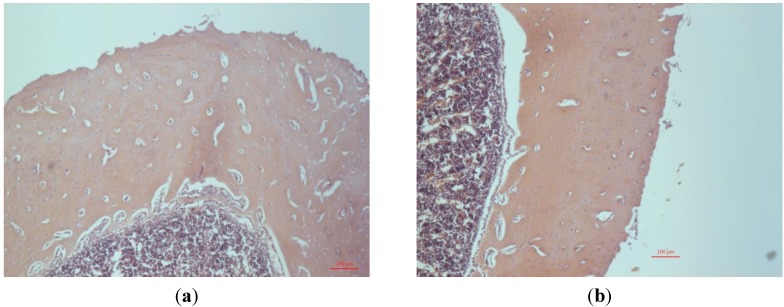
(**a**) Light microscopy image of a transverse section of rat tibia three weeks after injection of MgO paste. Primary objective magnification ×5. Htx-Eosin staining. (**b**) Light microscopy image of a transverse section of rat tibia three weeks after sham operation.

Examination, by light microscopy, of the sectioned tibia, showed a 25% increase in thickness of the compact bone in animals implanted with MgO-paste during three weeks of healing compared with sham-operated controls (Figure 1). An active formation of compact bone by dense layers of osteoblasts is seen at the endosteal membrane of Mg-treated animals (Figure 1a) but not in sham-operated controls (Figure 1b). No effect was observed regarding the mass of trabecular bone. This response to MgO occurs later in the healing process since no difference in the thickness of compact bone was seen after two weeks of implantation (not shown).

It was then considered of interest to study the atomic (EDX) and molecular (ToF-SIMS) composition of the tibia sections of exposed animals and controls after two weeks of healing, when the thickness of compact bone did not differ between Mg-exposed animals and sham-operated controls.

**Figure 2:**
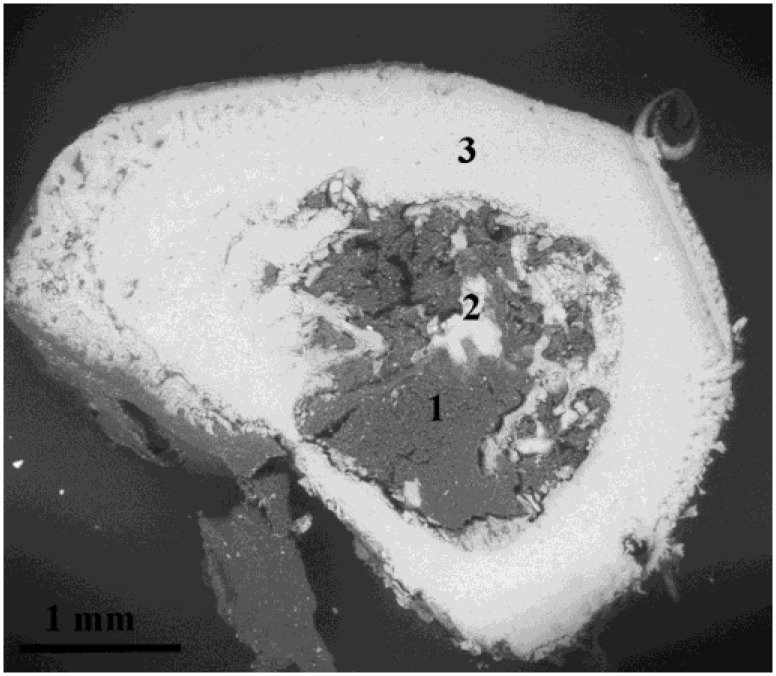
Environmental scanning electron microscopy (ESEM) backscattered electron image of a section of rat tibial bone, at two weeks after implantation of MgO in the bone marrow. The areas 1–3 correspond to the location of the EDX analysis, representing bone marrow, trabecular bone, and compact bone.

An environmental scanning electron microscopy (ESEM) backscattered electron image of sectioned rat tibia bone exposed to MgO for two weeks is shown in Figure 2. The bright areas represent bone tissue and the grey areas represent bone marrow. Remodeling of callus bone can be seen at the periosteal and endosteal membranes. Scattered trabecular bone extends into the bone marrow cavity.

EDX analysis shows the presence of Mg in the bone marrow, endostal bone and the surrounding compact bone (Figure 2). The content of atomic species in different areas of the bone cross section is shown in Table 1 and Table 2. The content of Mg in Mg-exposed bone does not differ from the corresponding values found in sham-operated controls. However, the content of calcium in the bone tissue is lower in bone from Mg-exposed animals than in sham-operated controls. Thus, the Mg/Ca ratio is higher in the Mg-exposed animal. The content of other atomic species related to bone minerals, oxygen and phosphorus, is also somewhat lower in the Mg-exposed animals compared to controls. Thus, the results obtained indicate an altered structure of the mineral phase as a result of Mg-exposure.

**Figure 3:**
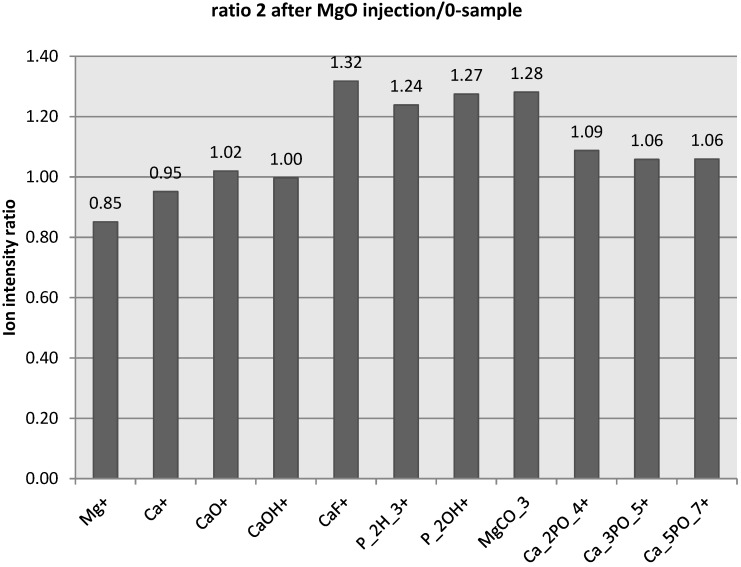
Ion intensity ratio between bone two weeks after injection of MgO-paste and control-sample. Ratios are an average of five areas 105 μm × 105 μm from each sample measured from the edge of the bone marrow cavity into the cortical bone.

The ToF-SIMS measurement showed elemental and molecular information measured in regions from the bone marrow into the cortical bone. Figure 3 shows the ratio of ion intensities between bone two weeks after MgO-injection and the control-sample bone. As with the EDX measurements bone exposed to Mg showed lower relative Ca^+^ levels. The Mg exposed bone instead showed higher intensities of several hydroxyapatite-related ion species [10,11], such as [Ca_2_PO_4_]^+^, [Ca_3_PO_5_]^+^ and [Ca_5_PO_7_]^+^, as well as other Ca and P related species such as [CaF]^+^ and [P_2_OH]^+^. The relative level of [MgCO_3_] was also found to be higher in the Mg-exposed bone. However, only the differences in [CaF]^+^ and [P_2_OH]^+^ ion intensities was statistically significant (students *t*-test, *p* < 0.05).

**Table 1:** Atomic species in different areas of Mg-exposed tibia bone (wt%).

Area/Atom	C	N	O	Na	Mg	P	Cl	K	Ca
Marrow	51.17	14.28	30.46	0.21	0.06	1.77	0.05	0.38	1.62
Endosteal	28.71	5.85	39.71	0.38	0.38	9.00	0.16	0.28	15.52
Compact	25.49	5.44	40.89	0.41	0.37	9.92	0.13	0.10	17.26

**Table 2:** Atomic species in different areas of tibia bone of sham-operated controls (wt%).

Area/Atom	C	N	O	Na	Mg	P	Cl	K	Ca
Marrow	53.51	14.38	26.75	0.22	0.08	2.32	0.04	0.42	2.28
Endosteal	25.7	0.09	42.05	0.43	0.37	11.39	0.16	0.31	19.67
Compact	24.52	0.33	41.89	0.41	0.38	11.92	0.17	0.24	20.80

## 4. Discussion

A rapidly increasing interest in Mg as a resorbable implant material is seen in recent publications and several studies have been published on the effects of Mg on bone healing and bone formation rate [10,11,12]. The results of these studies indicate that implanted Mg induces an increase in the amount of bone surrounding the implants, but, as to the difference between callus bone, remodeling bone and compact bone, only minor attention has been given. To the best of our knowledge, this is the first study describing the isolated effect of Mg on the thickness of compact bone.

Bone remodeling in response to implantation of MgO in rabbits has been reported previously [13], showing that MgO accelerates the remodeling process. This finding has been reproduced also in the rat model (data not shown). The present study was conducted in order to find out more about the effects of MgO implantation after the remodeling phase. The similarity between the processes of bone remodeling during fracture healing and bone healing at implants in rat tibia has been described previously [14,15]. In rat tibia, callus bone developed from the endosteum, already, at four days after injury, and remodeling of the callus was seen in the second week after injury [14]. In the sham operated rats the entire marrow cavity was cleared from callus bone during remodeling at four weeks after surgery. Thus, the bone samples used in the present study, taken three weeks after implantation, represent the diaphyseal part of rat tibia in a late stage of, or after, bone remodeling. The bone mass of the samples is contained mainly in cortical bone with a few trabeculae (Figure 1). The increased thickness of the compact bone, induced by MgO implantation into the bone marrow, seems to occur within the time period between two and three weeks after surgery. During the first two weeks after implantation, the Mg is obviously redistributed from the bone marrow probably to the bone mineral. The composition of the bone mineral seems to alter during this process.

The effect of Mg on the bone mass of compact bone, reported here, has been reported previously by Zierold [7], who showed an increased X-ray density of the entire tibia where Mg was implanted. The effect of Mg on bone X-ray density was not commented on in the paper, but is clearly seen in the X-ray image (Figure 2D in [7]). In addition, in some experiments made to exploit the bone formation induced by Mg upon implant healing, using porous titanium implants covered with Mg, more mineralized bone was found surrounding Mg-coated implants than around porous titanium implants [16].

The inclusion of MgO into hydroxyapatite may be understood in parallel with the natural process of biomineralization, where mineral is initially deposited as amorpous CaPO_4_ along with large amounts of CaCO_3_. When inserting a paste of MgO into the bone marrow, MgOH will rapidly form and transform into MgCO_3_ which may be built into carbonated hydroxy-apatite. The results of the present report indicate an altered mineral structure of the bone of Mg-exposed animals. ToF-SIMS analysis revealed higher levels of MgCO_3_ and CaF in the Mg-exposed bone samples. Such changes in the mineral composition may eventually affect cellular processes, such as osteoclast mediated bone resorption.

Since the effect of Mg on the bone mass is not seen until three weeks after surgery, the process of formation of compact bone is a slow process and several possible mechanisms may be suggested. Cell activity can be changed early after implantation, showing its effect as an altered response to such environmental effects as mechanical load or hormones. Recent data suggest that bone adaptation and maturation may result in specific increase of the mass of compact bone and trabecular bone respectively, including mechanical load [17], estrogen receptor activation [18,19,20], and parathormone [21]. In this context, implantation of Mg may be added to the list of stimuli that induce a specific increase in the thickness of compact bone.

The mechanisms of specific increase in thickness of compact bone mass, in general, are not fully understood. The Mg-induced increase of compact bone thickness, reported here, may be due to activation of mechanoreceptors on osteocytes, activation of estrogen receptors, activation of metabolic enzymes, activation of sensory neurons, inhibition of apoptosis of osteocytes, or inhibition of bone resorption by Mg incorporated into the bone mineral. However, the long time span between stimulus and effect rules out the possibility of a direct effect of Mg on osteoblast or osteoclast activities, due to the limited life span of these cells.

In conclusion: Implantation of Mg into rat tibial bone defects increases compact bone thickness significantly, indicating a stimulatory effect of Mg, the detailed mechanism of which is in need of further research.

## 5. Conclusions

Implantation of MgO into rat tibia increases the thickness of compact bone, without affecting trabecular bone. The bone response is seen late in the remodeling process, when implanted MgO cannot be detected by EDX. The mineral composition of the bone was altered during remodeling as seen by ToF-SIMS analysis.
